# When chest pain is not what it seems: time for right diagnosis and right treatment—a case report

**DOI:** 10.1093/ehjcr/ytaf410

**Published:** 2025-09-03

**Authors:** Giulia La Vecchia, Ludovica Leo, Antonio Maria Leone, Rocco A Montone

**Affiliations:** Department of Cardiovascular and Pulmonary Sciences, Catholic University of the Sacred Heart, Largo Francesco Vito, 1, Rome 00168, Italy; Center of Excellence in Cardiovascular Sciences, Isola Tiberina Hospital-Gemelli Isola, Via Ponte Quattri Capi 39, Rome 00186, Italy; Department of Cardiovascular and Pulmonary Sciences, Catholic University of the Sacred Heart, Largo Francesco Vito, 1, Rome 00168, Italy; Department of Cardiovascular Sciences, Fondazione Policlinico Universitario A. Gemelli IRCCS, L.go A. Gemelli 1, Rome 00168, Italy; Center of Excellence in Cardiovascular Sciences, Isola Tiberina Hospital-Gemelli Isola, Via Ponte Quattri Capi 39, Rome 00186, Italy; Department of Cardiovascular Sciences, Fondazione Policlinico Universitario A. Gemelli IRCCS, L.go A. Gemelli 1, Rome 00168, Italy

**Keywords:** ANOCA, Sensitive heart, Chest pain, Case report

## Abstract

**Background:**

Chest pain is a common reason for emergency department (ED) visits, yet not all cases are attributable to coronary artery disease (CAD). The 2024 European Society of Cardiology (ESC) guidelines emphasize the importance of invasive coronary function testing in patients with angina and non-obstructive coronary arteries. Understanding alternative causes of chest pain is crucial for appropriate diagnosis and management.

**Case summary:**

A 58-year-old woman with hypertension, prediabetes, and a history of Takotsubo Syndrome presented with recurrent chest pain episodes, prompting multiple ED visits. Initial cardiac evaluations, including electrocardiogram (ECG), troponin levels, and ECG stress testing, were unremarkable. Repeated invasive coronary angiography (ICA) with a full physiological assessment ruled out obstructive CAD, microvascular dysfunction, and coronary vasospasm, suggesting a ‘sensitive heart syndrome’. Further evaluation revealed a spinal schwannoma at the thoracic level, compressing the anterior spinal roots. Neuropathic chest pain was confirmed, and treatment with pregabalin led to symptom relief.

**Discussion:**

This case highlights the importance of a structured stepwise diagnostic approach to chest pain. When cardiac causes are excluded, alternative diagnoses such as neuropathic pain should be considered.

Learning points

- Angina with non-obstructive coronary artery disease (ANOCA) requires a structured stepwise diagnostic approach, including a ‘full physiology’ evaluation of the coronary arteries to define the specific endotype of ANOCA.
- When cardiac causes are excluded, alternative causes of chest pain as neuropathic pain should be considered.

## Introduction

Angina with no obstructive coronary arteries (ANOCA) refers to several functional disorders of the coronary circulation in the absence of obstructive coronary artery disease (CAD) (any epicardial coronary artery stenosis <50%).^1^ Angina with no obstructive coronary arteries is a working diagnosis requiring a standardized stepwise approach with coronary functional and provocative testing to define the specific endotype underlying the clinical condition.^2^ It also highlights the need to consider non-cardiac causes of chest pain when invasive evaluations yield normal results. Non-cardiac causes of chest pain is a prevalent condition, ranging ∼13% of all cases of chest pain, with a significant impact on healthcare-related costs and quality of life (QoL).^3,4^ Given its heterogeneous nature—ranging from musculoskeletal and gastrointestinal disorders to neurological conditions—an accurate diagnosis requires a comprehensive work-up. Identifying the underlying aetiology is crucial to prevent unnecessary cardiac interventions and ensure appropriate treatment.

## Summary figure

**Figure:**
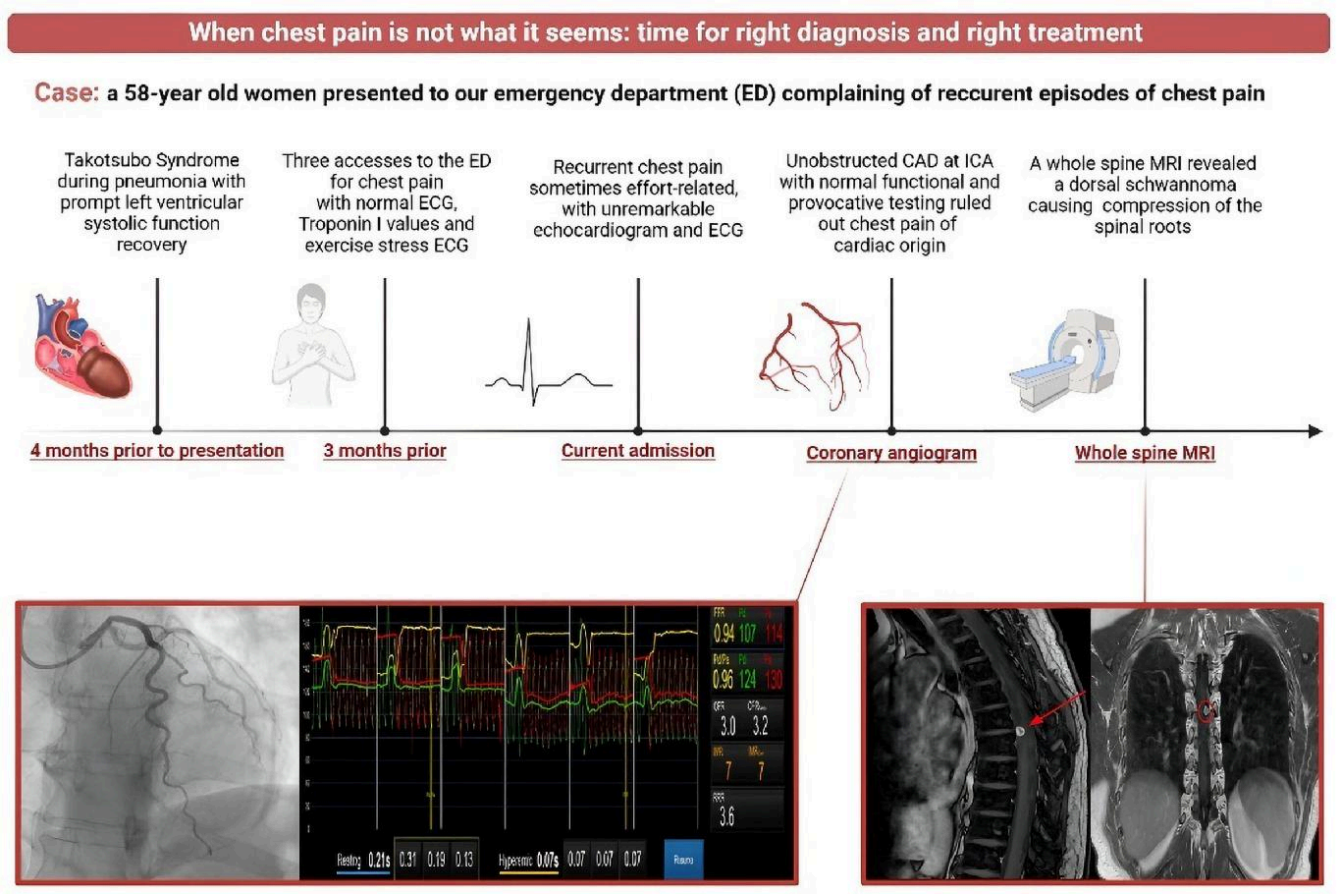


The figure illustrates the timeline of the case report and main findings. This figure was created with Biorender.com.

## Case presentation

A 58-year-old woman was referred to our attention for recurrent episodes of non-radiating chest pain, described as oppressive or piercing, sometimes associated with exertion, sometimes present at rest. The chest pain accompanied by unusually high levels of blood pressure prompted three accesses to the emergency department (ED) over the previous 3 months, none of which required hospital admission, as high-sensitivity troponin I levels (40, 35, and 32 ng/L; normal < 57 ng/L), electrocardiogram (ECG), and exercise stress ECG were unremarkable. The patient had a history of systemic arterial hypertension, along with overweight (body mass index of 29 kg/m^2^) and prediabetes with insulin resistance. Approximately 1 month before her first ED admission, she developed Takotsubo Syndrome (TTS) during pneumonia due to bronchiectasis. The diagnosis of TTS was confirmed by the evidence of unobstructed coronary arteries at invasive coronary angiography (ICA) and by cardiovascular magnetic resonance and transthoracic echocardiogram, showing a typical ‘apical ballooning’ pattern, with a prompt recovery of the left ventricular (LV) systolic function within 1 week from the symptoms onset. Her medications included perindopril/indapamide 10/2.5 mg/day, bisoprolol 1.25 mg/day, ranolazine 375 mg twice a day, metformin 500 mg twice a day, and liraglutide 3 mg subcutaneously per day.

**Figure 1:**
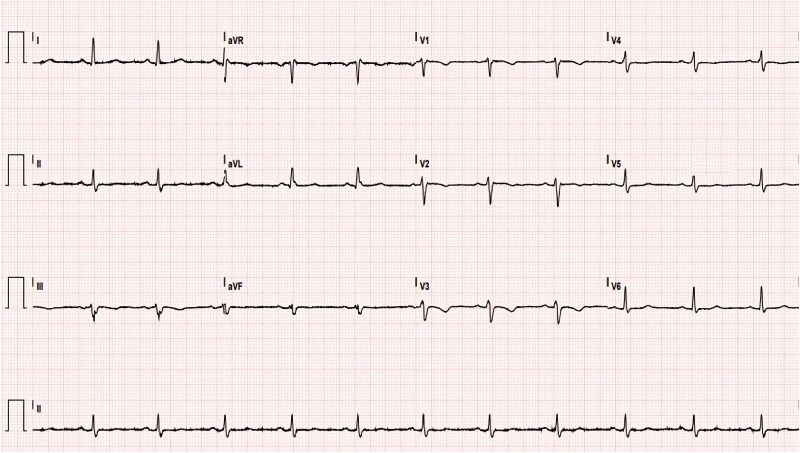
Electrocardiogram showing sinus rhythm without ischaemic changes.

Upon presentation, a cardiovascular physical examination revealed no pathological findings. The heart rate was 68 b.p.m., blood pressure was 140/100 mmHg, and oxygen saturation was 97%. Electrocardiogram showed sinus rhythm without ischaemic changes (*Figure 1*). Transthoracic echocardiogram demonstrated a left ventricular ejection fraction of 57% without regional wall motion abnormalities or pericardial effusion.

The initial treatment strategy focused on risk factors management, particularly hypertension, by replacing bisoprolol with carvedilol. Due to intolerance to carvedilol, the initial dose of bisoprolol was up-titrated, and amlodipine was introduced. However, there was no improvement in chest pain. A screening for pheochromocytoma was also performed to rule out secondary hypertension causes and returned negative results.

**Figure 2:**
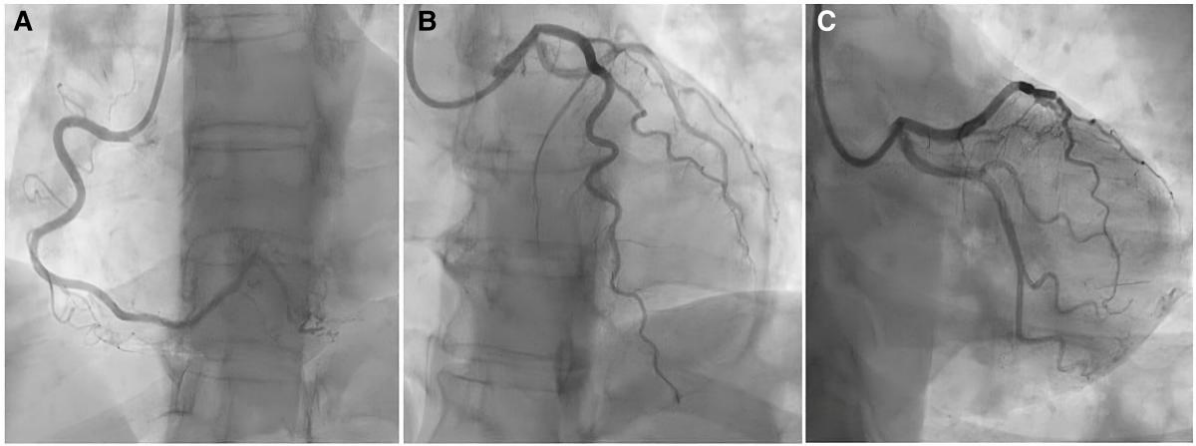
Invasive coronary angiography showing unobstructed coronary artery disease: (*A*) left anterior oblique 60°, showing the right coronary artery; (*B*) left anterior oblique 0° plus cranial 30°, displaying left anterior descending artery and diagonal branches; (*C*) right anterior oblique 20° plus caudal 20°, showing left circumflex coronary artery and obtuse marginal branches. LAO, left anterior oblique; CRA, cranial; RAO, right anterior oblique; CAU, caudal.

**Figure 3:**
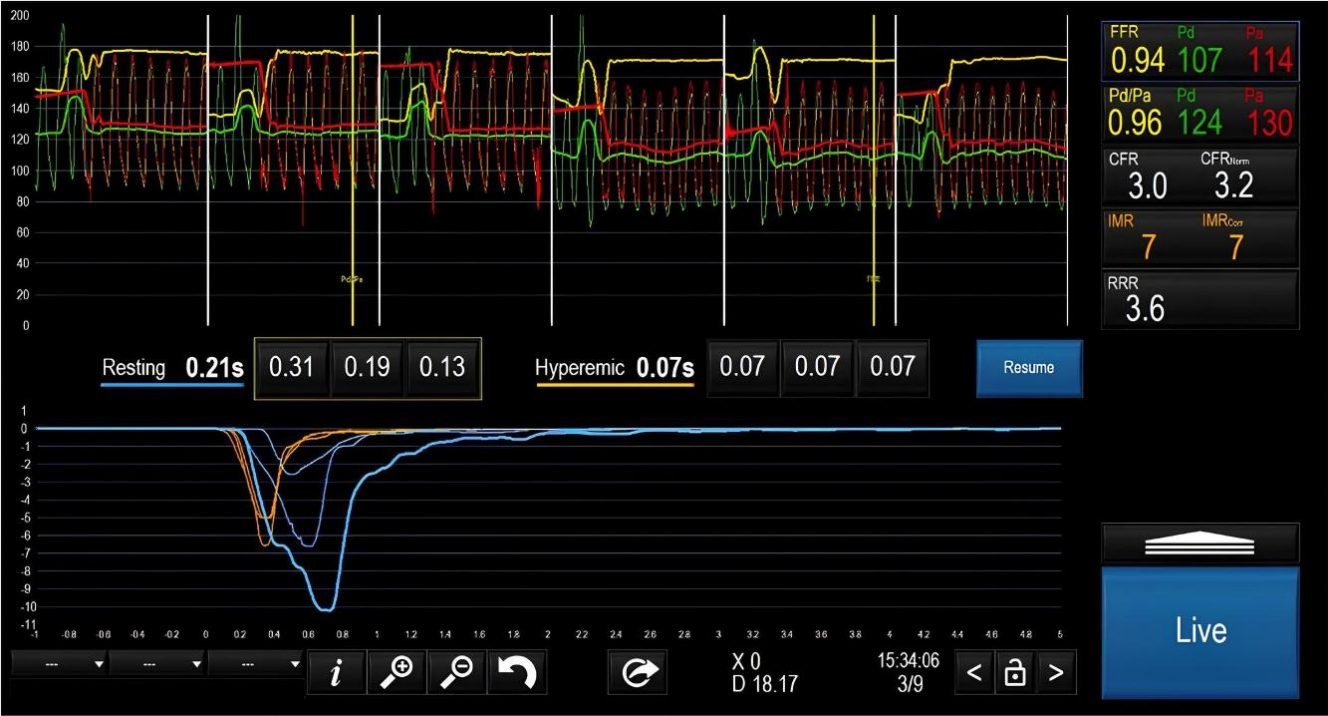
Invasive functional assessment demonstrating normal epicardial coronary artery coronary without microvascular dysfunction (fractional flow reserve 0.94, coronary flow reserve 3, index of microvascular resistance 7). CFR, coronary flow reserve; FFR, fractional flow reserve; IMR, index of microvascular resistance.

Therefore, ICA was repeated, confirming non-obstructive CAD (*Figure 2*). Hence, we performed a ‘full physiology’ evaluation of coronary circulation during adenosine and acetylcholine (ACh) infusions. Coronary function testing (CFT) returned normal values: a fractional flow reserve (FFR) of 0.94 excluded haemodynamically significant stenosis, while a coronary flow reserve (CFR) of 3.0 and an index of microvascular resistance (IMR) of 7 excluded coronary microvascular dysfunction (CMD) (*Figure 3*). Stepwise intracoronary infusion of 20, 50, and 100 mcg of ACh in the left coronary artery (LCA) revealed no epicardial nor microvascular coronary vasospasm. At the same time, she experienced chest pain during intracoronary adenosine, ACh, and contrast medium administration. The clinical presentation and the coronary angiogram were consistent with a ‘sensitive heart syndrome’. Hence, the patient was reassured, emphasizing that her coronary arteries were normal.

**Figure 4:**
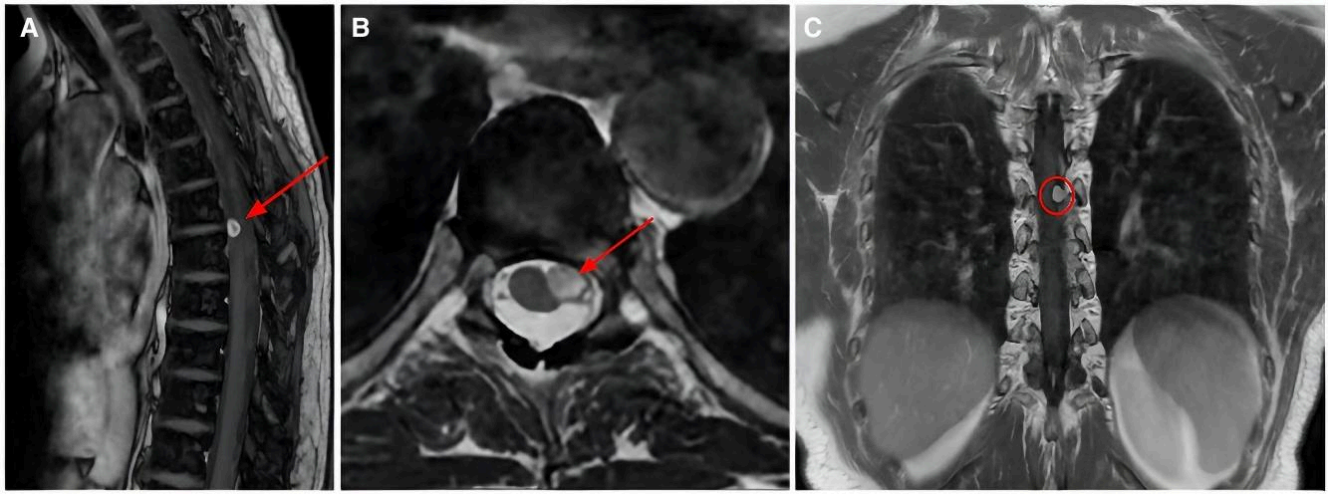
Magnetic resonance imaging of the thoracic spine. Axial (*A* with an arrow), sagittal (*B* with an arrow), and coronal (*C* within the circle) T2-weighted images reveal the presence of a left-lateral D6–D7 extramedullary intradural expansive lesion, consistent with a schwannoma. MRI, magnetic resonance imaging.

Afterwards, the chest pain recurred, along with cervical pain, fatigue, and burning paresthesia in the left dorsal region and ipsilateral arm. The patient also reported the simultaneous appearance of a skin lesion suspicious for a tick bite. These findings required laboratory and radiological assessments to rule out infectious or systemic diseases that may affect the peripheral nervous system. A whole spine magnetic resonance imaging (MRI) revealed a small intradural extramedullary lesion in the dorsal spine (D6–D7), compatible with a benign nerve sheath tumour. The diagnostic work-up was completed with a neurological evaluation, neurophysiological studies, and a thoracic spine MRI at 3 months follow-up, which confirmed the presence of the D6–D7 expansive lesion causing a compression of the anterior spinal roots, consistent with a spinal schwannoma (*Figure 4*). Considering the potential neuropathic origin of the patient’s chest pain, pregabalin 75 mg/day was prescribed. The patient was also treated with selective nerve root blocks, leading to symptom relief. At 1-year follow-up, she is awaiting for spine surgery.

## Discussion

Chest pain is responsible for ∼5.5% of all visits to the ED, with significant healthcare-related costs and impact on QoL.^5,6^ However, not all cases of chest pain are related to potentially life-threatening conditions such as CAD.

The Coronary Microvascular Angina (CorMicA) study investigated the application of stratified medicine in patients with ischaemia and unobstructed coronary artery disease (INOCA) and demonstrated that 11% of these patients have chest pain of non-cardiac origin.^7^

Non-cardiac chest pain is an exclusion diagnosis that requires a stepwise standardized diagnostic work-up.^5^ Firstly, obstructive CAD must be excluded by ICA or CCTA. In persistently symptomatic patients despite optimal medical treatment, the 2024 ESC guidelines for the management of chronic coronary syndromes stressed the importance of performing invasive coronary functional testing to further assess the specific aetiology of ANOCA/INOCA (Class I level of recommendation B).^8^ Hence, normal invasive physiology needs to be demonstrated during CFT (FFR > 0.80, CFR ≥ 2.0, IMR ≤ 25) to confirm the absence of obstructive CAD and exclude coronary microvascular dysfunction. Finally, Ach vasoreactivity testing is required to rule out epicardial or microvascular vasospasm.^5^

In our clinical case, the patient underwent ICA with a ‘full physiology’ approach study that ruled out any cardiac cause of chest pain and revealed a suspicion of a ‘sensitive heart syndrome’.^1,2^ ‘Sensitive heart syndrome’ refers to a condition where patients experience chest pain not linked to structural heart disease.^1^ It is due to an abnormal autonomic response and increased pain perception, often triggered by emotional or physical stress.

In our patient, a comprehensive diagnostic work-up for extracardiac causes of chest pain pointed out a possible neuropathic origin due to a spinal neurinoma, also known as schwannoma. Schwannomas are benign nerve sheath tumours within the spinal canal, primarily localized in the lumbar region.^9^ Spinal cord compression can trigger symptoms in the corresponding dermatome. Our patient’s space-occupying lesion was localized at the D6–D7 level and involved the anterior spinal roots, resulting in radicular chest pain and paresthesia. In particular, given that the D6–D7 spinal roots correspond to the dermatomes at the inferior border of the sternum, their involvement may have mimicked angina pectoris. Accordingly, a targeted treatment with pregabalin, which specifically acts on neuropathic pain by modulating the release of several excitatory neurotransmitters at the level of the central nervous system, led to an effective improvement in the patient’s symptoms.

## Conclusion

Chest pain must be considered heart-related until proven otherwise. The diagnostic approach to patients with ANOCA requires a precision medicine strategy. This includes a comprehensive assessment with CFT. Once cardiac disease has been ruled out, further investigation of alternative causes of chest pain is pivotal to identifying the right diagnosis and initiating the right treatment.

## Lead author biography


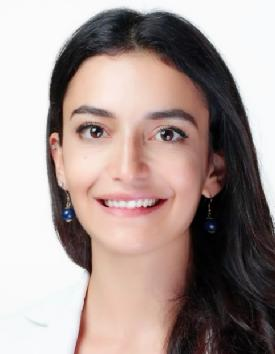


Giulia La Vecchia obtained her medical degree in 2018 at the University of La Sapienza in Rome, Italy. She completed her Cardiology residency at the Catholic University of Sacred Heart in Rome, Italy. She is currently attending her PhD program at the Catholic University of Sacred Heart in Rome.

**Consent:** The authors confirmed that appropriate informed consent was obtained from the patient for submission and publication of this case report in accordance with the Committee on Publication Ethics (COPE) guidelines.

## Data Availability

A data availability statement cannot be shared publicly due to patient confidentiality and privacy concerns. Data may be available upon reasonable request to the corresponding author.
